# Subarachnoid Neurocysticercosis Presenting as a New-Onset Seizure in an Immigrant From Guatemala

**DOI:** 10.7759/cureus.18241

**Published:** 2021-09-24

**Authors:** Shane Clark, Rodrigo F Alcala, Nelson A Luque, Norman Beatty

**Affiliations:** 1 Department of Medicine, University of Florida College of Medicine, Gainesville, USA; 2 Division of Infectious Diseases and Global Medicine, University of Florida Health, Gainesville, USA

**Keywords:** scolex, mri brain and spine, parasitic disease, taenia solium, subarachnoid neurocysticercosis

## Abstract

Subarachnoid involvement is a rare but severe form of neurocysticercosis (NCC), leading to serious complications if not recognized and treated appropriately. Imaging of the brain usually involves computed tomography and/or magnetic resonance image (MRI) of the brain, both of which can lead to a diagnosis of NCC. We present a 20-year-old female with no significant past medical history presenting with a new-onset seizure whose clinical treatment was significantly altered when subarachnoid involvement was identified. This case highlights the importance of brain MRI in recognizing subarachnoid disease, an important subset of NCC disease presentation.

## Introduction

Neurocysticercosis (NCC) is an infection of the central nervous system by the cysts of the cestode, *Taenia solium* [1]. It is thought to be one of the most common causes of seizure disorder worldwide, especially in endemic areas such as South America, sub-Saharan Africa, and parts of Asia [2,3]. While transmission of *T. solium* cysts is a rare occurrence in the United States (U.S.), travelers from endemic regions still account for greater than 2,000 cases per year in the U.S. [4]. Treatment of NCC can be challenging due to the costs associated with antiparasitic treatment and associated neurological sequelae requiring intervention. NCC is thought to burden the U.S. healthcare system by over $100 million per year, but more research is needed to better understand these costs [5]. Increased healthcare disparities also exist among the populations most at-risk for this disease in the U.S. As of 2015, cysticercosis is only a reportable disease in Arizona, California, New Mexico, Oregon, Texas, and Alaska. Currently, there is no nationwide reporting system of suspected or confirmed cases of cysticercosis and/or NCC [5].

There are two types of infections that can be caused by the *T. solium* species. The first, taeniasis is when a person is infected by larval cysts that develop into the adult tapeworm and the second cysticercosis, is when a person ingests the organism’s eggs which invade the body as larval cyst [1]. When cysticercosis reaches any part of the central nervous system this is termed NCC. Subarachnoid NCC represents a particularly severe form of the disease [6]. Most subarachnoid NCC patients present with multiple cysticerci that can be located in subarachnoid space and rarely in the brain's ventricles [1]. Clinical manifestations depend on the location of the cyst but can include seizures, hydrocephalus, meningeal signs, and even focal neurological deficits [1]. Here we present a case of successfully treated subarachnoid NCC at our institution.

## Case presentation

A 20-year-old female who is otherwise healthy and has no significant past medical history presented to our institution as a hospital-to-hospital transfer following a new-onset seizure. The patient was in her usual state of health until the night before admission when she started complaining of dizziness, malaise, and blurry vision. The next morning several family members witnessed acute seizure-like activity at home which lasted approximately five minutes. Emergency medical services were called and she was brought to a regional medical center. The patient did not remember arriving at the hospital or the seizure event. She was born in a rural setting in Guatemala and emigrated to the U.S. four years ago. She now works as a potato farmer in Putnam County, Florida. She denied any alcohol or recreational substance use. In the emergency department, the patient was somnolent but otherwise hemodynamically stable. Urine drug screening was negative. A lumbar puncture was performed and cerebrospinal fluid (CSF) analysis was significant for clear fluid, elevated white blood cells (22 cells/mm^3^), which were 98% lymphocytes, and elevated total protein (76 mg/dL). Gram stain of CSF revealed the presence of polyclonal appearing lymphocytes but no organisms were seen. Aerobic and anaerobic CSF cultures were negative for any growth. Magnetic resonance imaging (MRI) with contrast revealed enhancing cystic lesions in the parenchyma with surrounding edema. Levetiracetam was initiated for antiepileptic treatment and she was transferred to our institution for infectious diseases and neurosurgical consultation.

At our institution, the patient denied any past seizures, headache, fever, chills, night sweats, vision changes, or cough. Her neurological exam showed no acute abnormalities. Complete blood count (CBC) was notable for leukocytosis 10.7x10^3^/mm^3^ with a neutrophil predominance with no eosinophilia. The complete metabolic panel was normal. Screening for human immunodeficiency virus, *Mycobacterium tuberculosis* (QuantiFERON gold plus), Toxoplasma gondii, and Chagas disease was negative. Ophthalmology was consulted and did not find any ocular abnormalities. Cysticercosis ELISA antibody in serum was negative.

**Figure 1:**
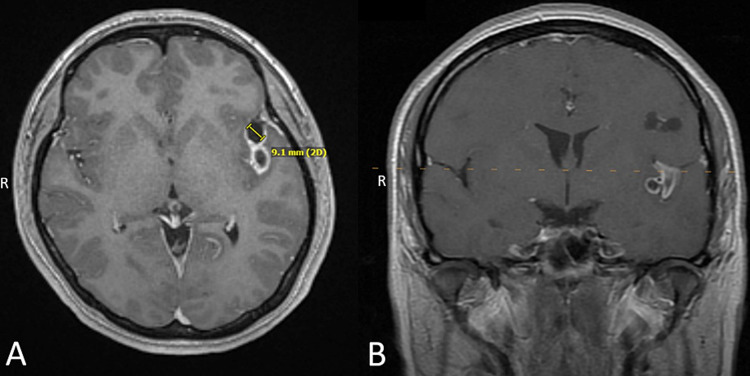
Complex cysticerci cysts located in the left Sylvian fissure between the temporal and parietal lobes (A – axial; B – coronal) within the subarachnoid space.

**Figure 2:**
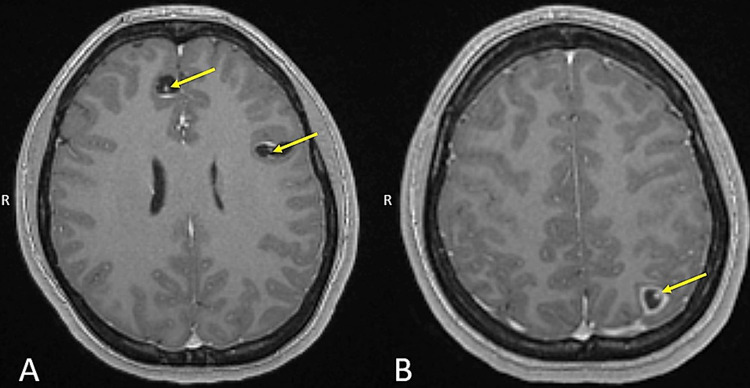
Scolex (yellow arrows) demonstrated in cysticerci cysts in the right frontal and left parietal lobes (A) and left parietal lobe (B).

A repeat MRI with and without contrast was done at our institution and detected a total of six enhancing cystic lesions. Four in the left cerebral hemisphere and two in the right cerebral hemisphere. One of the lesions located in the left Sylvian fissure of the temporal region and located in the subarachnoid space close to the middle cerebral artery (MCA) showing associated inflammation in MCA branches (Figures 1A, 1B). All the cystic lesions on the repeat MRI revealed central dots concerning the scolex (Figures 2A, 2B).

**Figure 3:**
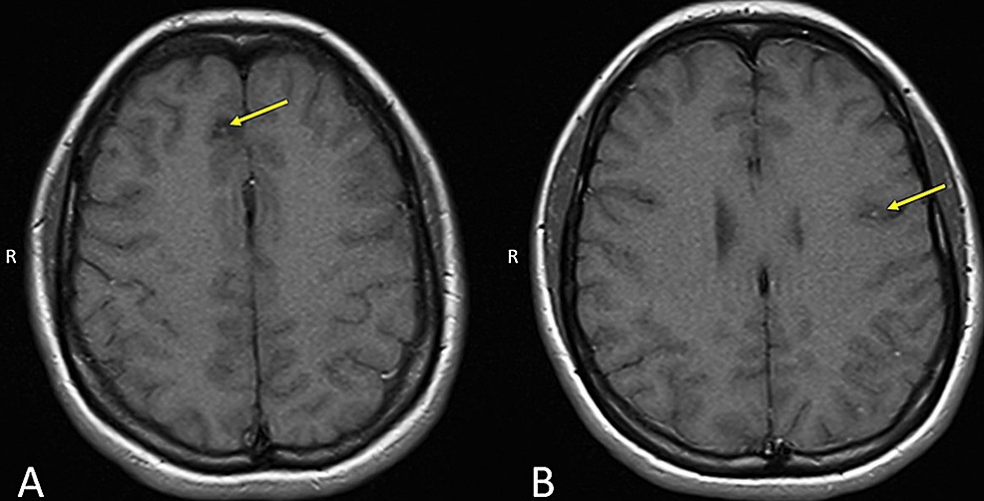
Compared to initial MRI findings (Figure 2), all six cysticerci on repeat MRI displayed a decrease in overall size and contrast enhancement after initiating antiparasitic treatment. This can be seen here in the right frontal (A) and left parietal lobes (B) cysticerci (yellow arrows).

Treatment was initiated due to pathognomonic epidemiological, clinical, and radiologic findings consistent with a diagnosis of NCC with subarachnoid involvement. The case was reviewed with the Department of Health and Human Services National Institutes of Health NCC research team who agreed with the radiological findings. Levetiracetam was continued for antiepileptic treatment and a 14-day course of albendazole (500mg twice a day by mouth), praziquantel (1,200mg twice a day by mouth), as well as dexamethasone (2mg every eight hours by mouth) were initiated. A repeat MRI was completed three weeks after initiation of antiparasitic therapy and revealed decreased size of all cysticerci and reduced contrast enhancement (Figures 3A, 3B). Due to the subarachnoid involvement, a prolonged treatment course was warranted and the patient was referred for ongoing care and management with the National Institutes of Health in Bethesda, Maryland for continued management.

## Discussion

NCC is considered to be one of the most common parasite infections involving the central nervous system worldwide, but it is still quite rare in the U.S. [2]. It has a long latency period, between months to decades as was seen in our patient who moved to the U.S. four years prior to presentation, and therefore exposure history should not be limited to recent events [7]. After a thorough medical and social history, brain imaging is recommended in all patients, with the first line being computed tomography (CT). If a scolex is identified on imaging as was seen in our case, this is pathognomonic for NCC, and treatment should be initiated [7]. The additional use of MRI is also recommended due to its sensitivity in diagnosing the scolex, small parenchymal lesions, posterior fossa lesions, or recognizing involvement of the subarachnoid space [8].

If imaging results are suspicious for subarachnoid involvement, it is particularly important to treat this condition aggressively because severe complications can occur in this subset of patients due to the cyst’s proximity to blood vessels [7]. Primarily, the perivascular inflammation, which was also seen in our patient, can lead to severe complications including endarteritis, inflammatory aneurysms, or thrombosis [9]. Subarachnoid involvement can also lead to further manifestations which depend on the location of the lesions but symptoms can include cognitive dysfunction, hydrocephalus, or chronic meningitis [10]. As was seen in our patient, performing a follow-up MRI was crucial in diagnosing subarachnoid involvement and drastically changed our patient’s subsequent medical care.

 If the diagnosis remains uncertain after imaging, cysticercosis serologies can be obtained. The test of choice is testing for antibodies using enzyme-linked immunotransfer blot (EITB) for parasite glycoproteins [11] with 86% sensitivity for NCC compared to 41% with enzyme-linked immunosorbent assay (ELISA) [12]. The sensitivity varies depending on the type of NCC, but it has shown promise in evaluating the treatment response in subarachnoid cysts [11,13]. In this case, the ELISA-based testing at a commercial laboratory was negative but given the known lack of sensitivity, this is to be expected. Providers may need to seek a tissue biopsy in some circumstances.

Once NCC is suspected, an early ophthalmology consult should be considered. Fundoscopy examination prior to initiation of anthelmintic treatment is strongly recommended in all patients to check for intraocular involvement because it can lead to severe complications including blindness [7]. This was performed successfully in our patient who did not have any ocular involvement. Treatment of NCC is becoming a greater problem in the U.S. with increased cost and unavailability of the necessary antiparasitic medications [4]. Longer courses of antiparasitic medication than the normal 10-14 days in the parenchymal form of the illness have shown benefit in patients with subarachnoid involvement [14]. The duration of treatment can vary depending on clinical improvement and MRI findings [7]. Furthermore, dual therapy with praziquantel (50mg/kg/day) and albendazole (15mg/kg/day) has been showing benefit with severe parenchymal disease and is now recommended for subarachnoid presentations [7]. With these recommendations, our patient was treated with both antiparasitic medications with an anticipated extended course for at least one year.

In addition to antiparasitic medications, anti-inflammatory agents such as steroids have been shown to lessen the inflammation caused by initiating antiparasitic therapy, and steroids are recommended in all patients with subarachnoid cysts [7,15]. Due to evidence showing recurrent seizures in NCC patients, antiepileptic medications are also recommended [7]. Both of these agents were used in conjunction with antiparasitic medications in our patient and seemed to help improve her condition as evidenced by repeat MRI showing reduced cyst size and no further breakthrough seizures prior to discharge from the hospital.

## Conclusions

Our patient presented with a new-onset seizure with radiologic evidence of subarachnoid NCC. Among those who have emigrated from endemic regions of cysticercosis to the U.S. with new-onset seizures, NCC should be considered and CT and/or MRI brain should be done to help investigate this neglected tropical disease. Subarachnoid NCC requires prolonged treatment and management to avoid complications that can occur such as communicating hydrocephalus, stroke, meningitis, and breakthrough seizures.
